# Systemic hypoferremia and severity of hypoxemic respiratory failure in COVID-19

**DOI:** 10.1186/s13054-020-03051-w

**Published:** 2020-06-09

**Authors:** Akshay Shah, Joe N. Frost, Louise Aaron, Killian Donovan, Hal Drakesmith, Collaborators, Stuart R. McKechnie, Simon J. Stanworth

**Affiliations:** 1 Radcliffe Department of Medicine, University of Oxford and John Radcliffe Hospital, Level 4 Academic Block, Headley Way, Oxford, OX3 9DU UK; 2 MRC Human Immunology Unit, MRC Weatherall Institute of Molecular Medicine, University of Oxford, John Radcliffe Hospital, Oxford, UK; 3 Adult Intensive Care Unit, John Radcliffe Hospital, Oxford University Hospitals NHS Foundation Trust, Oxford, UK; 4 Haematology Theme, NIHR Oxford Biomedical Research Centre, Oxford, UK

Dear Editor,

Coronavirus disease 2019 (COVID-19) caused by severe acute respiratory coronavirus 2 (SARS-CoV-2) was declared a pandemic on March 11, 2020 [1]. Risk factors associated with respiratory failure in patients with COVID-19 include older age, neutrophilia and elevated inflammatory and coagulation markers [1]. Inflammation is often accompanied by systemic hypoferremia and low iron levels may impair hypoxia sensing and immunity [2], and increase the risk of thromboembolic complications [3]—which are all of significant concern in COVID-19. However, the iron status of COVID-19 patients is unclear. Therefore, we sought to characterise iron parameters, including serum iron, in COVID-19 intensive care unit (ICU) patients and relate these to disease severity.

## Methods

We retrospectively evaluated any serum iron profiles that were measured in critically ill patients with COVID-19 within 24 h of admission to the ICU, John Radcliffe Hospital, Oxford, UK, between March 31, 2020, and April 25, 2020. Relevant clinical and laboratory data were extracted from routine datasets. The number of patients who had died, had been discharged, and were still in ICU as of May 12, 2020 was recorded.

We stratified patients according to severity of hypoxemic respiratory failure on admission to ICU—severe (PaO_2_/FiO_2_ ratio < 100 mmHg) versus non-severe (PaO_2_/FiO_2_ ratio 100–300 mmHg). All patients with severe hypoxemia required invasive mechanical ventilation and prone positioning. Mann-Whitney rank sum test was used to compare nonparametric continuous variables between these two groups. All statistical tests were 2-tailed, and statistical significance was defined as *P* < .05. Analyses were performed using PRISM version 8 (GraphPad Software).

## Results

A total of 30 patients were included. Table 1 shows the demographic, clinical and laboratory characteristics of the included patients. Overall, 17 (57%) patients were male. The median (interquartile range (IQR)) age was 57 (52–64) years.

**Table 1:** Clinical, laboratory and iron profile characteristics of study cohort, total and stratified by severity of hypoxemia

Characteristic	All patients (*n* = 30)	Severe (*n* = 10)	Non-severe (*n* = 20)	
Age, median (IQR), years	57 (52–64)	57 (53–75)	57 (52–64)	0.949
Males, *n* (%)	17 (57)	5 (50)	12 (60)	
Females, *n* (%)	13 (33)	5 (50)	8 (40)	
APACHE II score, median (IQR)	13.0 (9.8–15)	14.5 (12–20)	13 (12–18)	0.7512
Clinical Frailty Scale, *n* (%)				
1	23 (77)	9 (90)	14 (70)	
2	4 (13)	1 (10)	3 (15)	
> 3	3 (10)	0	3 (15)	
Respiratory support, *n* (%)				
Non-invasive ventilation	18 (60)	7 (701)	11 (55)	0.509
Invasive ventilation	26 (87)	10 (100)	16 (80)	0.378
Prone position	17 (57)	10 (100)	7 (35)	0.004
Advanced cardiovascular support, *n* (%)	4 (13)	0 (0)	4 (20)	0.379
Advanced renal support, *n* (%)	10 (33)	5 (50)	5 (25)	0.271
PaO_2_/FiO_2_ ratio, median (IQR)	127.5 (87–200.6)	82.5 (77–87)	190.8 (127.5–277.5)	< 0.001
Laboratory data (normal range)				
Haemoglobin (g/L) (120–150), mean (SD)	130.4 (20.1)	124.7 (16.7)	133.2 (21.4)	0.280
White cell count (× 10^9^/L) (4.0–11.0), mean (SD)	10.6 (4.8)	11.0 (5)	10.4 (4.7)	0.733
Lymphocyte count (× 10^9^/L) (1.0–4.0), mean (SD)	0.74 (0.4)	0.50 (0.2)	0.87 (0.42)	0.015
D-dimer (μg/mL) (0–500), median (IQR)	3286 (1302–14,227)	9505.5 (845–5023)	2462 (1453–9850)	0.462
Fibrinogen (g/L) (1.5–4.0), median (IQR)	6 (5.5–6.3)	6 (5.5–6.3)	6.1 (5.5–6.4)	0.670
CRP (mg/L) (0–5), mean (SD)	246.2 (100.1)	235.8 (101.8)	251.4 (101.5)	0.69
Iron parameters (normal range)				
Ferritin (mcg/l) (10–200), median (IQR)	1476.1 (656.6–2698)	903.8 (566.9–2789.2)	1566.1 (729–2511.5)	0.569
Serum iron (μmol/L) (11–30), median (IQR)	3.6 (2.5–5)	2.3 (2.2–2.5)	4.3 (3.3–5.2)	< 0.001
Transferrin (g/L) (1.8–3.6), median (IQR)	1.5 (1.1–1.8)	1.3 (0.8–1.8)	1.5 (1.1–1.8)	0.784
Transferrin saturation (%) (16–50), median (IQR)	9 (7–13)	7 (6–12)	12 (8–14)	0.122
Pulmonary embolism, *n* (%)	16 (53.5)	7 (70)	9 (45)	0.203
Outcome as of 10 May 2020, *n* (%)				
Died in ICU	6 (20)	5 (50)	1 (5)	
Still alive in ICU	16 (53)	3 (30)	13 (65)	
Discharged alive from ICU	8 (27)	2 (20)	6 (30)	
ICU length of stay, median (IQR), days	8 (4–11)	7 (4–9)	9 (4–12)	

*Abbreviations*: *APACHEII* Acute Physiology and Chronic Health Evaluation II, *CRP* C-reactive protein, *ICU* intensive care unit, *IQR* interquartile range, *SD* standard deviation

**Fig. 1:**
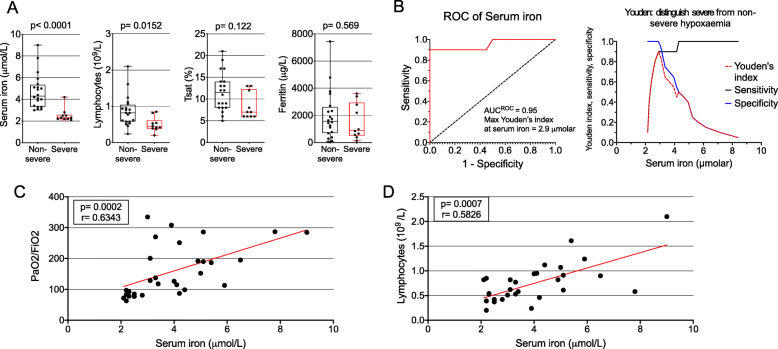
Associations between markers of iron status, lymphocyte count and severity of hypoxemia. **a** Boxplots show the 25th, 50th and 75th percentiles (box); 10th and 90th percentiles (whiskers); and data points (circles) of serum iron, transferrin saturation (Tsat) and serum ferritin, stratified by severity of hypoxemia. **b** Receiver operating characteristic (ROC) curve and Youden Index for serum iron in distinguishing severe and non-severe hypoxemia. **c** Correlation serum iron and PaO_2_/FiO_2_ ratio. **d** Correlation between serum iron and lymphocyte count

Compared with patients with non-severe hypoxemia, patients with severe hypoxemia had significantly lower levels of serum iron (median 2.3 (IQR, 2.2–2.5) vs 4.3 (IQR, 3.3–5.2) μmol/L, *p* < 0.001) and lymphocyte counts (mean (SD) 0.50 (0.2) vs. 0.87 (0.4), *p* = 0.0152). There were no statistically significant differences in transferrin saturation and serum ferritin levels between groups (Fig. 1a). The area under the curve for receiver operating characteristic curves for serum iron to identify severe hypoxemia was 0.95; the optimal Youden Index for distinguishing between severe and non-severe hypoxemia was a serum iron concentration of 2.9 μmol/L (sensitivity 0.9, specificity 1.0) (Fig. 1b). By linear regression, serum iron was associated with lymphocyte count and PaO_2_/FiO_2_ ratio (Fig. 1c, d). The proportion of patients with pulmonary emboli was numerically higher in patients with severe hypoxemia, but this was not statistically significant.

## Discussion

This is the first study describing iron status in COVID-19. Our data suggest that serum iron may be a useful biomarker for identifying disease severity in COVID-19, whilst also being a potential therapeutic target. Serum iron was lower when compared with other cohorts of non-COVID-19 ICU patients reported previously, including those with sepsis [4]. The association of serum iron with lymphocyte counts could reflect the requirement of the adaptive immune response for iron [5] and may contribute to possible T cell dysfunction reported in COVID-19 [6].

Hypoferremia is likely to be due at least in part to inflammation-driven increases in hepcidin concentrations [2]. Anti-inflammatory drugs such as tocilizumab will likely suppress hepcidin synthesis through inhibition of interleukin-6 (IL-6) [6] and so increase serum iron. Other potential therapeutic strategies include hepcidin antagonists and hypoxia-inducible factor inhibitors. Additionally, unlike hepcidin and IL-6, serum iron is measured widely and so could assist with identification and monitoring of severity of disease. Our results support performing a larger study to better characterise these patterns.

## Data Availability

The dataset used and analysed for this study are available from the corresponding author on reasonable request.

## Abbreviations

COVID-19: Coronavirus Disease-2019
FiO_2_: Fraction of inspired oxygen
ICU: Intensive care unit
IL-6: Interleukin-6
PaO_2_: Partial pressure of oxygen in arterial blood
SARS-CoV-2: Severe acute respiratory coronavirus 2
